# The effects of alpha lipoic acid on muscle strength recovery after a single and a short-term chronic supplementation - a study in healthy well-trained individuals after intensive resistance and endurance training

**DOI:** 10.1186/s12970-020-00389-y

**Published:** 2020-12-01

**Authors:** Eduard Isenmann, Lucas Trittel, Patrick Diel

**Affiliations:** 1 Institute for Cardiovascular Research and Sports Medicine, Department of Molecular and Cellular Sports Medicine, German Sports University, 50933 Cologne, Germany; 2 Department of Fitness and Health, IST-University of Applied Sciences, 40233 Dusseldorf, Germany

**Keywords:** Intensive training, Alpha lipoic acid, Recovery, Muscle damage, Inflammation, Performance

## Abstract

**Background:**

Alpha lipoic acid (ALA) has been demonstrated to have anti-inflammatory activity and was tested as a drug for the treatment of various diseases. ALA is also frequently used as a nutrition supplement, in healthy individuals or in competitive athletes. However, information from intervention studies investigating physiological effects of an ALA in athletes after exercise is limited. Therefore, the aim of this study was to investigate the effects of single and short-term chronic ALA supplementation on the muscle strength recovery and performance of athletes after intensive exercise.

**Methods:**

In a double-blind, randomised, controlled trial in cross-over design, 17 male resistance and endurance-experienced athletes successfully participated. The subjects were divided into two groups (ALA and Placebo) and underwent a standardized single training session and a high intense training week. At certain time points (T0, T1a (+ 3 h), T1b (+ 24 h) and T2 (+7d)) blood samples were taken and markers for muscle damage, inflammation and oxidative stress were investigated. In addition, the maximum performance in the back squat was measured at all time points.

**Results:**

In the chronic training experiment, a moderate inhibition of muscle damage and inflammation could be observed in the ALA-group. Performance in the back squat was significantly reduced in the placebo-group, but not in the ALA-group. No anti-oxidative effects could be observed.

**Conclusions:**

Our data indicate possible effects of ALA supplementation, during intensive training periods result in a reduction of muscle damage, inflammation and an increase of recovery. Whether ALA supplementation in general may enhance performance and the exact training / supplementation scenarios needs to be investigated in future studies.

**Supplementary Information:**

The online version contains supplementary material available at 10.1186/s12970-020-00389-y.

## Introduction

In competitive sports, the time span needed for muscle recovery plays a decisive role. The aim is to reduce this time period to a minimum to set new training impulses as quickly as possible. Recovery in sports is a multifactorial process that can be measured in sports medical biomarkers such as the kinetics of muscle damage or inflammatory processes and in applied sports science performance tests such as maximum strength tests [1–3].

After intensive concentric-eccentric training, the stressed muscles are damaged. This is initiating physiological restructuring processes in the body. Investigations have shown that after intensive strength training [4], endurance training (marathon or longer) [5], extended mountain running [6] and high-intensity interval training [7], training-induced muscle damage (EIMD) is present. Massive EIMD can be also observed after eccentric contractions against high loads [8–11]. Classical quantitative markers for muscle damage are the increase of creatine kinase (CK) and myoglobin (Myo) serum concentrations. EIMD results also in an inflammatory response of muscle tissue caused by the infiltration of macrophages and can be detected during the entire muscle recovery process [12]. This inflammatory response can be quantitatively analysed by measuring serum concentrations of different cytokines like tumor necrosis factor alpha, (TNF-α), interleukin 1ß, (IL-1ß) and interleukin 6, (IL-6) [13–15]. Consequently, due to muscle damage and inflammation, shifts in tissue fluids and swelling can occur in the training body regions [16]. In addition to muscle damage and inflammation, intense training can cause oxidative stress due to increased radical formation of hydrogen peroxide (H_2_O_2_), superoxide (O_2_^−^), hypochlorous acid (HOCl) and nitric oxide (NO^−^) [17]. To counteract an inflammatory response and oxidative stress, the supplementation of substances with anti-inflammatory and antioxidant capabilities is usually practiced after physical exercise. One of these substance is alpha lipoic acid (ALA) [18].

ALA has different functions in the human metabolism. ALA is part of the multienzyme complex of pyruvate dehydrogenase, alpha-ketoglutarate and branched alpha-keto acids [16–18]. Additionally, ALA has an antioxidant effect. ALA is able to recycle endogenous glutathione, one of the most important antioxidants. It is able also to act as a radical scavenger of hydroxy radicals, hypochlorous acids, peroxide radicals and singular oxygen, as well as to form chelate complexes with metal ions [19–23].

Antioxidant effects of ALA have been demonstrated in different clinical trials. In alzheimer patients ALA have neuroprotective effects of ALA [24]. Moreover, ALA protects against oxidative injuries in non-neuronal and neuronal tissue [25]. ALA, which is also involved in mitochondrial bioenergetic reactions, has attracted considerable attention as an antioxidant for the treatment of diabetic complications such as retinopathy, neuropathy and other vascular diseases [26, 27]. This has been demonstrated as well in animal models, but also in human intervention studies [28–30]. Interventions could showed that the use of ALA can reduce the concentration of oxidized LDL (oxLDL) in type 2 diabetes patients [31]. Furthermore, oxLDL is an important parameter in connection with other chronic diseases, oxidative stress and physical activity [32, 33].

Besides antioxidants effects, also anti-inflammatory effects of ALA have been described. ALA is able to inhibit the transduction factor nuclear factor kappa B (NFkB) by modulating mitogen-activated protein kinase (MAPK) via Inhibitor kappa B (IkB). NFkB is one of the main signalling pathways for the activation of inflammatory reactions. If NFkB is inhibited, inflammation can be reduced [34–38]. Anti- inflammatory activity such as inhibition of c reactive protein (CRP), TNF-α, IL-6, IL8 and IL-10 was observed in type 2 diabetics [39, 40]. Similar effects have been demonstrated in patients with metabolic syndrome [41]. In such patients also effects of ALA on body weight reduction and reduction of body mass index (BMI) are described, which further illustrates effects of ALA on energy metabolism [42, 43].

Even used frequently as a nutrition supplement, information regarding effects of ALA in healthy individuals or in competitive athletes is limited. In competitive sports, the use of protein and carbohydrates preparations against muscle damage and inflammation [44, 45] as well as vitamin C and E preparations as antioxidants is common [46]. Recently, it has been demonstrated that ALA reduces the serum concentrations of creatine kinase after a 90-min endurance load indicating possible protective effects with respect to muscle damage [18]. Data regarding potential protective or pro-regenerative effects of ALA are not available so far.

The aim of this study was therefore to determine effects of ALA supplementation on muscle recovery, inflammation and possible anti-oxidative activities after a one-time resistance training and after a six-day high-intensity training week in well-trained individuals. The specific research questions to be investigated are:

Does a single treatment with ALA after acute exercise increase the ability to recover?

Does a short-term chronic application of ALA during a high-intensity training period effect the ability to recover?

Does the application of ALA after acute and during chronic training protocol prevents the loss of performance?

## Materials and methods

### Participants

The study design was approved by the ethics committee of the German Sports University Cologne and is in accordance with the Declaration of Helsinki. It is registered in the German Register of Clinical Trials under the name EACSARKA and the registration number DRKS-ID: DRKS00018768.

All participants were informed about the study design and confirmed their voluntary participation in writing. Excluded from participating in the study were people who were injured, ill or who supplement any medications or dietary supplements. During the study, no further sports activities were allowed and the natural diet should not be changed. All participants were healthy male sports students of the German Sports University Cologne and were both strength- and endurance-experienced athletes. The athletes had minimum 2 years of strength training experience and at least a back squat performance of 1.5x of body weight. In endurance, they were able to run 10 k under 1 hour.

To confirm the requirements, the maximum strength was tested before the investigation and the running performance was checked by a successful participation in an official run (certificate must not be older than 1 year).

### Study design

**Fig. 1:**
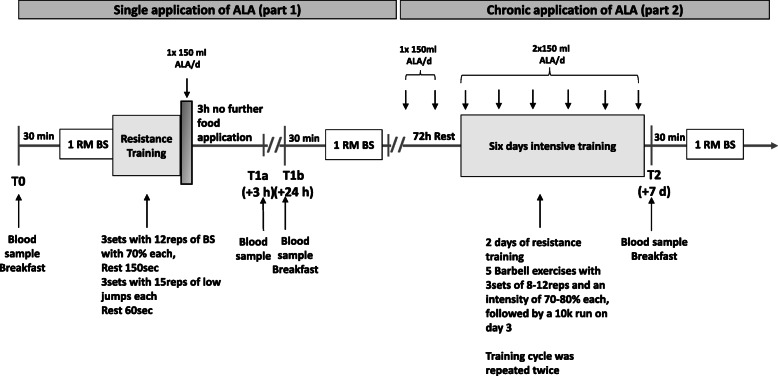
Study design

The study was carried out as a randomized, double-blind study in crossover design. Between the intervention phases there was a washout period of 4 weeks to counteract possible training adaptations. The intervention phase took place twice (ALA and PL supplementation) and was divided into two parts. During the first part, the single application of ALA was examined after intensive strength training. The initial examination was followed by a 72 h recovery period. Afterwards the second part of the examination was performed with a short-term chronic supplementation of ALA and a training protocol over 6 days. Finally, a further initial examination took place. The complete study design is shown *in* Fig. 1*.*

### Training protocol single application of ALA

All subjects came rested (48 h no training) and fasting (12 h without meal) in the morning for the initial examination. The anthropometric data were collected and a blood sample was taken (T0). Afterwards there was a standardized breakfast, which consisted in 60 g oat flakes, 5 g honey and a banana (115 g) as well as hot water. The nutritional values and calorie numbers are shown in Table 1*.*

**Table 1:** Standardized breakfast

Food	Quantity	Proteins	Carbohydrates	Fat	Calories
oat flakes (JA oat flakes)	60 g	8.1 g	35.2 g	4.2 g	224.4 kcal
banana (Chiquita)	115 g (one piece)	1.2 g	25.3 g	0.2 g	110.4 kcal
Honey (Langnese)	5 g	0 g	3.8 g	0 g	15 kcal
water	individual	/	/	/	/
**Total**		**9.1 g**	**54.3 g**	**4.4 g**	**349.8 kcal**

After 30 min of digestion, the warm up started with 5 min of running and 10 min of specific movement for the lower body. Following, the maximum strength performance in back squat was tested. The test protocol was based on the guidelines of the NSCA for performance testing [3]. Immediately afterwards the load protocol was carried out. This includes three sets of squats with 12 repetitions, an intensity of 70% of 1RM and a set pause of 150 s. In addition, after the squats, three sets of low jumps (combination of drop and counter movement jumps) were performed, each with 15 repetitions and a 60-s pause. Subsequently, the volunteers received a drink consisting of 200 ml water and 3 ml solubilisat (150 mg ALA or a placebo). The ALA and placebo bottles were made specifically for this study (Athenion GmbH, Berlin, Germany). There was no difference in taste or appearance between the two beverages. Only the screw cap was considered a distinguishing feature (red/white cap).

In the 3 hours after the intervention, further feeding was prohibited (only water was allowed). After 3 h another blood sample (T1a, + 3 h) was taken and the subjects were allowed to eat until 8 pm. On the following day, a blood sample was taken again (T1b, + 24 h), the standardized breakfast and the maximum strength test in back squat were performed.

### Six days training protocol and short-term chronic supplementation of ALA

**Fig. 2:**
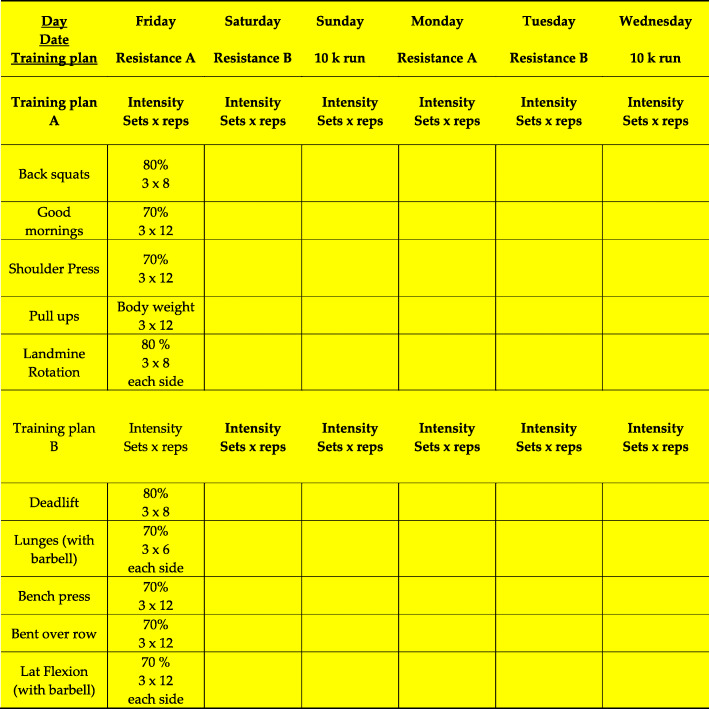
Six days training protocol

After 72 h recovery, the 6 days training protocol started. It included four strength training session with two different training plans. Both plans included five barbell exercises with three sets, 8–12 reps and an intensity of 70–80%. The exercise selection, sequence and intensity are based on a previous investigation [47]. After two strength training sessions followed a 10 km run with a maximum training duration of 60 min. This training cycle of two resistance and one endurance sessions was carried out 2 times in 6 days. The complete training plan is shown in Fig. 2. The second run was followed by the second initial examination (T2, + 7 d).

### ALA supplementation

As described in previous sections, in the first part of the study, only a single application of ALA (150 mg) was used. ALA or placebo was supplemented once a day on the two rest days between the two training protocols. During the 6 days, subjects supplemented ALA/PL once before the training session (2 h before) and immediately after the training session (in total 300 mg of ALA).

### Measurements

#### Skeletal muscle Creatine kinase (CK) and myoglobin (Myo)

Skeletal muscle specific creatine kinase activity (CK) and myoglobin (Myo) concentrations in the serum were determined using the COBAS h 232 Point-of-Care-System (Roche Diagnostic Systems, Rotkreuz, Switzerland) at T0 and T1b (+ 24 h) and T2 (+ 7d).

### Serum cytokine levels

IL-6 concentrations of serum samples were analysed using the Human IL-6 ELISA Kit High. Sensitivity (Abcam, Cambridge, United Kingdom). IL-10 serum concentrations were analysed using a human IL-10. ELISA Kit (Abcam, Cambridge, United Kingdom). IL-6 and IL-10 concentrations were determined at T0, T1a (+ 3 h) and T2 (+ 7 d).

### Serum HDL, LDL and oxidative LDL levels

OxLDL concentrations of serum samples were analysed using the Human oxLDL/MDA Adduct Elisa. (Bensheim, Germany). OxLDL concentrations were determined at T0 and T2 (+ 7 d). LDL and HDL concentration were determined using the Beckman Coulter AU analyzers (Inc., 250 S. Kraemer Blvd. Brea, CA 92821, USA). LDL and HDL concentrations were determined at T0 and T2 (+ 7 d).

### Strength performance – 1RM back squat

The strength test was performed at T0, T1b (+ 24 h) and T2 (+ 7 d). The strength protocol is based on the guidelines of NSCA [3]. All subjects performed three warm-up sets and started at 50% of their estimated maximum power with 10 repetitions. In the following warm-up sets, the weight was increased by about 10–20% and the repetition number reduced to five and three repetitions. This was followed by four 1RM tests, starting with about 90% of the estimated 1RM. Between the sets a four-minute break was taken.

### Statistical analyses

For the statistical analyses the current version of SPSS (IBM SPSS Statistics 26.0, Ehningen, Germany) was used. The results were presented as mean values with standard deviations (SD). For all parameters, a Z transformation was performed at each time points to analyse possible outliers. Values above a triple standard deviation were not taken into account in the analysis. All measurement parameters were tested for normal distribution with Kolmogorov-Smirnov test. Subsequently, with a two way Ankova with repeated measurement and Bonferroni test, time and time*group effects were analysed for 1RM back squat. At all biomarkers, a Mann Whitney U and Kruskal Wallis Test were used. Significant differences are set at *p* < .05 and marked with * (time effect) and # (time*group effect). In addition, Cohen’s d with pooled standard deviation was used to calculate the effect size between the groups at T2 (+7d). The images were created using GraphPad PRISM software (GraphPad 8.0 Software, Inc. La Jolla, CA, USA).

## Results

Seventeen out of 18 volunteers successfully participated in the study. One subject was unable to complete the study for health reasons. The subjects were 23.5 (SD 3.1) years old, 183.06 cm (SD 6.8) tall and weighed 81.5 kg (SD 7.4) at the beginning of the study. The weight was additionally measured after 7 days (T2) and at the beginning and end of the second intervention phase. All anthropometric data are shown in Table 2.

**Table 2:** Anthropometric data of all participants

	Intervention 1		Intervention 2		ALA		Placebo	
	T0	T2 (+7d)	T0	T2 (+7d)	T0	T2 (+7d)	T0	T2 (+7d)
Age (in y)	23.47 SD 3.14	iT0	iT0	iT0	iT0	iT0	-iT0	iT0
Height (in cm)	183.06 SD 6.77	iT0	iT0	iT0	iT0	iT0	iT0	iT0
Weight (in kg)	81.56 SD 7.41	82.12 SD 7.70	81.99 SD 7.94	82.78 SD 7.94	82.13 SD 7.93	82.71 SD 8.34	81.43 SD 7.59	82.19 SD 7.56

### Effects of ALA on leg strength - 1RM Back squat (1RM BS)

A direct parameter to quantify muscle strength recovery is testing of skeletal muscle strength at different time points after exercise. In our experimental design leg strength was measured by the 1RM in back squat at the time points T0, T1b and T2.

After a single training load between T0 and T1b no significant decrease of 1RM BS (*n* = 17) could be observed in both groups. The 1RM of the PL- group decreased from 128.2 kg (SD 30.3) to 125.3 kg (SD 28.0) (*p* = .051). In the ALA-group, a similar decrease of power could be observed. Performance decreased from 128.8 kg (SD 27.9) to 125.9 kg (SD 29.3) (*p* = .053). There was no time*group difference (*p* = .992).

**Fig. 3:**
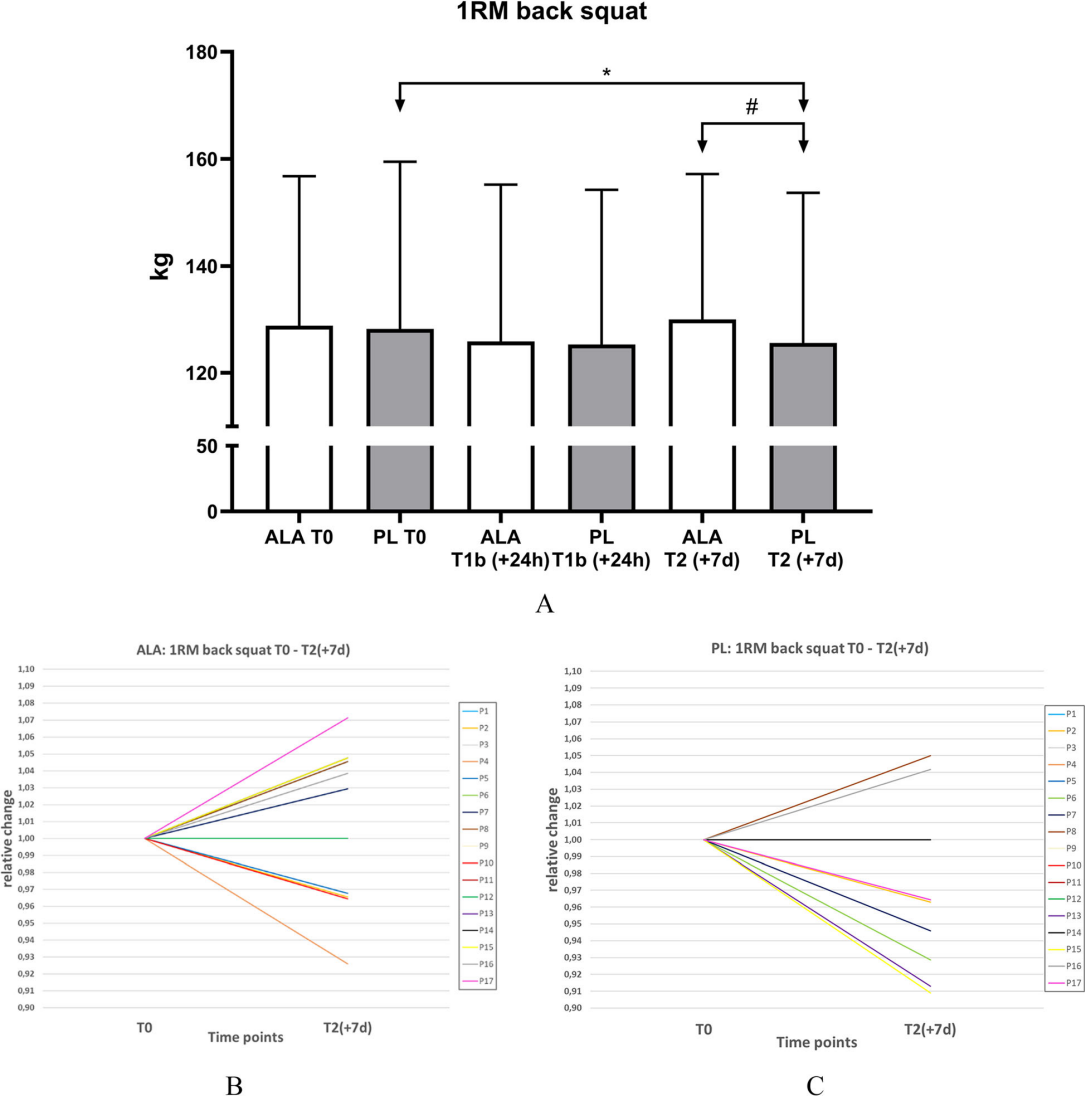
**a** 1RM back squat, **b** individual change ALA-group, **c** individual change PL-group. Time effects were marked with *; Time x group effects were marked with #

After 6 days of training, both a time and a time*group effect (*p* = .028#, d = .164) could be observed. In contrast to the PL, whose performance was 125.6 kg (SD 27.2) (*p* = .032*), the ALA group was able to maintain its maximum strength at the baseline (T0) of 130.0 kg (SD 27.2) (*p* = .320). The changes in 1RM BS of ALA and PL group at T0, T1b and T2 are shown in Fig. 3a.

A total of eight subjects (P1, P7, P8, P13, P14, P15, P16, P17) with ALA supplementation improved their performance after the six-day protocol. Five subjects were able to maintain their performance (P3, P6, P9, P11, P12) and only four subjects (P2, P4, P5, P10) had a lower performance at time T2 (+7d) (Fig. 3b). Due to the fact, that some of the subjects had the same percentage change (highlighted in colour in Table 3A, supplementary material), only ten progressions can be seen. In contrast, only two increases (P8, P16) were observed in the back squat in the placebo group (Fig. 3c). Nine subjects (P1, P3, P4, P5, P9, P10, P11, P12, P14) were able to maintain their performance and six subjects (P2, P6, P7, P13, P15, P17) had a decrease in performance. The PL group also shows the same percentage changes (highlighted in colour in Table 3B, supplementary material), which means that only nine progressions can be seen.

### Effects of ALA on skeletal muscle damage

To investigate effects of ALA on training induced skeletal muscle damage serum concentrations of CK and Myo were determined after a single training load and a repeated training load for 6 days.

**Fig. 4:**
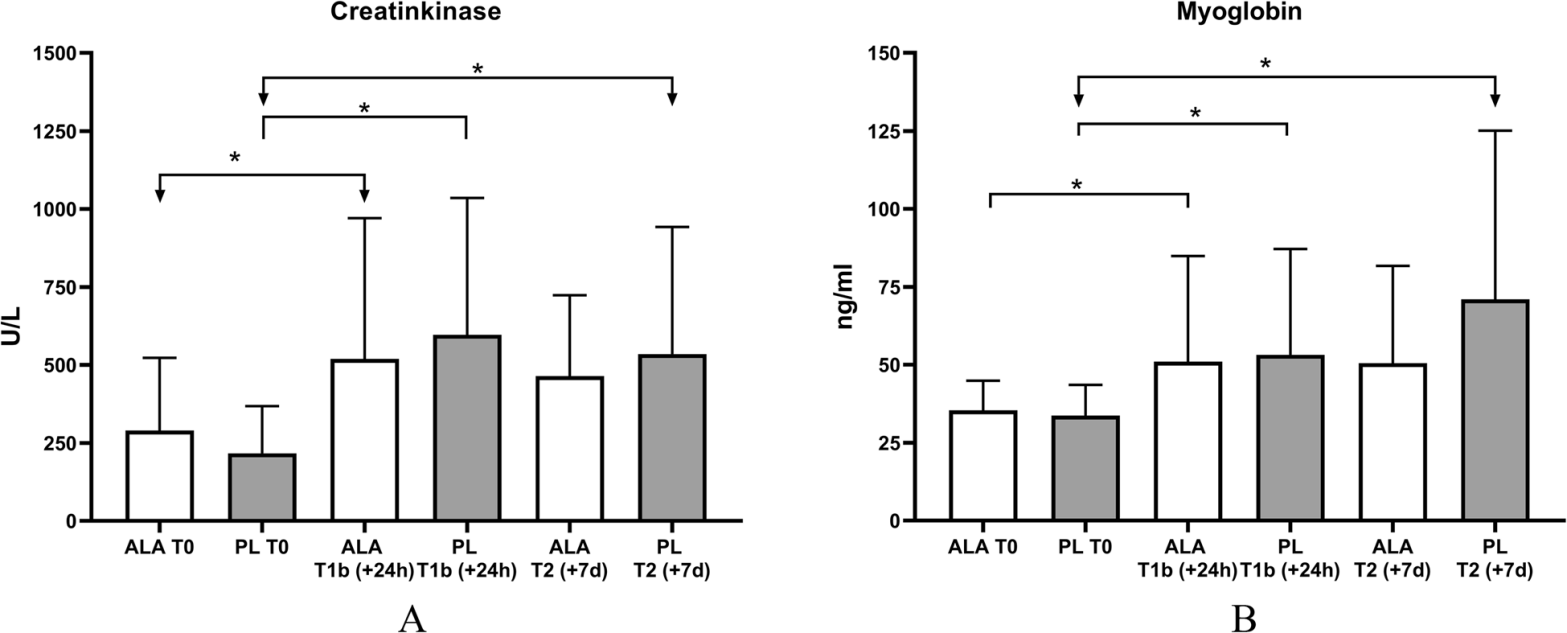
**a** Creatinkinase, **b** Myoglobin. Time effects were marked with *; Time x group effects were marked with #

Twenty-four hours after the single training load (T1b), CK (*n* = 14) serum concentrations increased significantly in all groups (T1) (PL: *p* = .000*, ALA: *p* = .014*). There was an increase in the ALA group from 290.6 U/L (SD 232.1) to 519.0 U/L (SD 436.5) compared to 217.1 U/L (SD 146.1) to 597.0 U/L (SD 422.5) in the PL-group. There was no significant time*group difference at T1b (*p* = .119) (Fig. 4a). Similar changes could be observed for Myo serum concentrations (*n* = 16). In both groups, the myoglobin concentration increased significantly (PL: *p* = .002*, ALA: *p* = .045*) after the single training session. In the ALA-group, the value increased from 35.4 ng/ml (SD 9.1) at T0 to 51.0 ng/ml (SD 32.9) at T1b. The PL group increased from 33.8 ng/ml (SD 9.4) at T0 to 53.2 ng/ml (SD 32.9) at T1b. There was no significant time *group difference at T1b (*p* = .581) (Fig. 4b).

After the six-day repeated training load, in the PL-group CK serum concentrations increased significantly from 217.1 U/L (SD 146.1) at T0 to 534.6 U/L (SD 392.6) at T2 (*p* = .005*). In the ALA-group, however, there was no significant increase in CK (T0: 290.6 U/L (SD 232.1); T2: 464.1 U/L (SD 250.3); *p* = .426). Similar effects could be observed for Myo. Only in the PL-group the Myo concentration increased significantly from 33.8 ng/ml (SD 9.4) at T0 to 71.06 ng/ml (SD 52.4) at T2 (Fig. 4b) (*p* = .008*). In the ALA-group no significant increase could be observed (from 35.4 ng/ml (SD 9.1) at T0 to only 50.5 ng/ml (SD 30.3) at T2 (*p* = .059). There were no significant time*group differences at T2, neither for CK (*p* = .136, d = .212) nor for Myo (*p* = .130, d = .467) serum concentrations. The changes in CK and Myo of the two groups at different times are shown in Fig. 4 a/b*.*

### Effects of ALA on training induced inflammatory response

To get insights into the physiological mechanisms after physical activity, serum concentrations of the pro-inflammatory cytokine IL-6 (*n* = 16) and the anti-inflammatory cytokine IL-10 (*n* = 17) were determined at T0, T1a and T2 in addition to the serum concentrations of the pro-inflammatory cytokine IL-6 (n = 16).

**Fig. 5:**
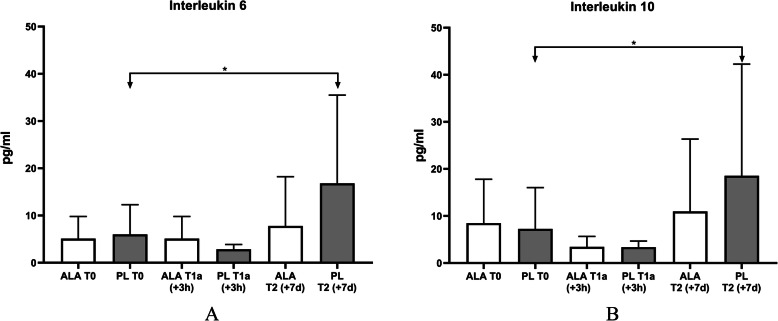
**a** Interleukin 6, **b** Interleukin 10. Time effects were marked with *; Time x group effects were marked with #

A significant increase of IL-6 and IL-10 serum concentrations between T0 and T2 could be observed in the PL-group. In the PL-group the IL-6 concentration increased from 6.0 pg/ml (SD 6.1) to 16.8 pg/ml (SD 18.1) (*p* = .005*). However, such an increase was not detectable in the ALA-group. IL-6 increased from 5.1 pg/ml (SD 4.7) to 7.8 pg/ml (SD 10.4) (*p* = .249). A time*group effect could not be observed (*p* = .107, d = .587). A similar increase could also be observed with IL-10. In the PL-group the concentration increased from 7.2 pg/ml (SD 8.5) to 18.6 pg/ml (SD 23.0) (*p* = .035*). In the ALA-group the value changed from 8.5 pg/ml (SD 9.3) to 10.2 pg/ml (SD 14.5) (*p* = .554). A time*group effect could not be observed (*p* = .959, d = .428). The changes in IL-6 and IL-10 of all groups at T0 and T2 are shown in Fig. 5 a/b*.*

At T1a no significant change was observed in either group for IL-6 and IL-10 (IL-6: PL: .05, ALA: .092; IL-10: PL: .289, ALA: .136). There was also no detectable time*group effect at T1a for both parameters (IL-6: *p* = .795; IL-10: *p* = .479).

### Effects of ALA on markers for oxidative stress

**Fig. 6:**
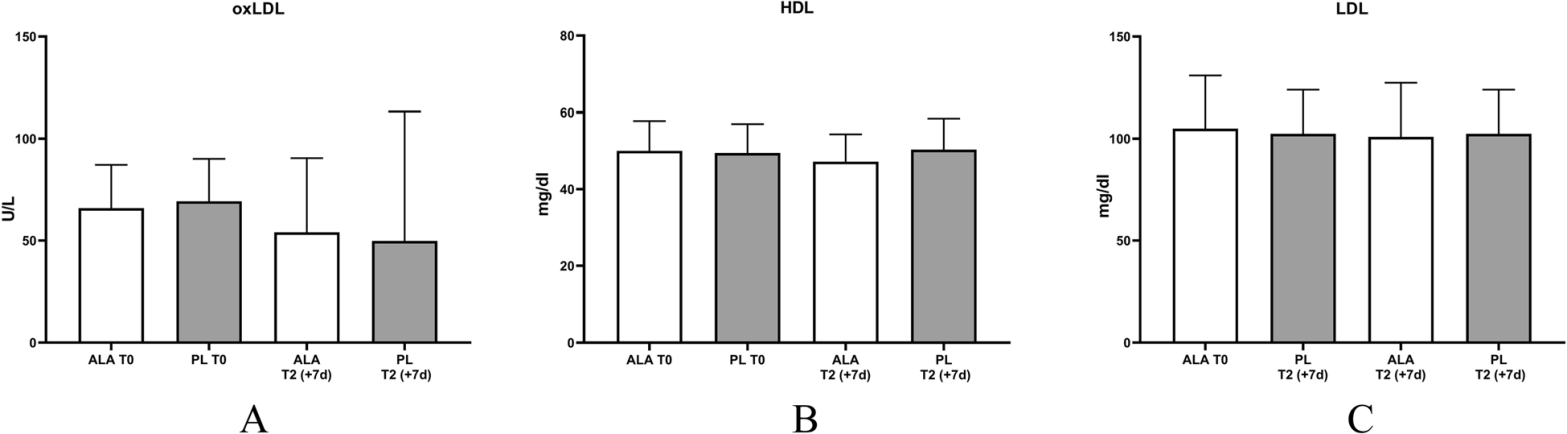
**a** oxLDL, **b** HDL, **c** LDL. Time effects were marked with *; Time x group effects were marked with #

ALA has been described to have strong anti-oxidative activity. Therefore, the concentrations of oxLDL, as oxidative stress marker, were analysed in the serum at T0 and T2. No significant changes between T0 and T2 could be observed (Fig. 6a).

In addition to oxLDL, general lipoprotein values (HDL and LDL) were measured. No significant changes were found in HDL and LDL (Fig. 6b/c). In addition, all results are summarized in Table 4 (Supplementary Materials).

## Discussion

The aim of this study was to investigate whether the application of ALA after acute and during chronic training protocols prevents the training induced loss of performance in athletes.

As a functional parameter for pro-muscle recovery effects of ALA supplementation the individual changes in 1RM BS, were investigated and operationalised. As shown in Fig. 3 the six-day chronic training protocol resulted in a reduction of leg strength, which could be counteracted by ALA supplementation. Even if only a time and time x group difference and no group difference could be observed at time T2 (+ 7 d), a interaction and marginal effect could be observed at time T2 (+ 7 d). This can be taken as an indication for possible pro-recovery effects. Similar effects have been demonstrated for other dietary supplements such as creatine or protein shakes [45, 48, 49].

So far, possible pro-recovery effects after training with ALA supplementation have not been described in the literature. Recent investigations have shown that the application of protein carbohydrate combinations counteracts the training induced loss of performance when administered a single application directly after exercise [45, 50, 51]. This could not be observed in this study for ALA (Fig. 3). Neither the ALA-group, nor the control group experience a significant reduce of performance in the acute training protocol. This may be due to the very high performance level of the participants, which is higher compared to the participants examined in the previous study [45]. However, the significance was only slightly missed (ALA: *p* = .053; PL: *p* = .051). Athletes who usually complete five training sessions per week and regularly perform back squats will need a higher load than three sets with 12 repetitions and 70% of the 1 RM as well as three sets with 15 low jumps. Nevertheless, it is not possible to draw conclusions about recovery based solely on the slight effect on the performance and short-term chronic ALA application. The change in performance must be considered in the context of the implied muscle damage and inflammatory response and the effect of ALA application. To investigate the effects of ALA supplementation on skeletal muscle damage in this study two independent parameters, CK and Myo concentrations in response to training, were measured. Both training protocols, the single training load and the six-days repeated training load protocol resulted in a significant increase of serum CK and Myo concentrations, indication skeletal muscle damage (Fig. 4) [8, 52–54].

As visible in Fig. 4a and b, a short-term chronic supplementation of ALA seems to have a small to moderate positive protective effect on skeletal muscle damage as indicated by our biomarkers (CK: d =. 212, Myo: d = .467). However, it is known that CK serum concentration is a very influenceable parameter and should be used with caution. Therefore, in our study only 14 subjects were considered for evaluation of this parameter. Two further subjects were declared as high responders and were individually evaluated. All individuals whose standard deviation was greater than 3.5 after the Z-transformation at T2 were declared as high responders. In general, high responders are individuals who react very strongly to a training stimulus and therefore do not reflect the general course of certain parameters [55]. Interestingly also in these individuals a clear effect of ALA on CK concentration can be seen (supplemental material, Fig. 4 A2). In addition, it also needs to be highlighted, that a possible protective effect ALA with respect to muscle damage has been also demonstrated by a second independent parameter, Myo (Fig. 4b). Similar to the 1-RM BS, no group difference could be detected at time T2 (+ 7 d). Nevertheless, we believe that due to the similar course and effects in CK, Myo concentration and BS, ALA has a reducing effect on muscle damage in chronic application. These observations are consistent with Morawin et al. who observe similar effects of 10 days ALA supplementation on CK after endurance training [15]. Effects of ALA supplementation on serum CK concentrations have also been shown in disease models, suggesting that ALA has a reducing effect on CK concentration [51].

To investigate the effects of ALA supplementation on training induced inflammation the cytokine serum concentrations of IL-6 and IL-10 were measured as biomarkers. It has been already shown that ALA inhibits the activity of the transcription factor NFkB, resulting in a reduced secretion of pro-inflammatory cytokines such as IL-6 [20, 34]. After the intensive chronic training protocol in our study, we observed that serum concentrations of cytokines, associated with inflammation, are modulated by ALA supplementation. After 6 days of intensive training, the IL-6 concentration in the placebo group increased significantly compared to the ALA group (Fig. 5). This confirms the assumption that ALA may suppress training-induced inflammation. Consequently, no anti-inflammatory response is activated in the ALA group (Fig. 5). After the single training program and application, no significant increase in cytokines was observed in both groups. These even decreased slightly at the time of measurement. This may be due to a possible activation of the cytokines in the tissue and not in the measured blood serum, so that the possible measurement time was not chosen appropriately in this design. The assumption that a reaction can be observed after three hours of exposure is based on various previous studies [45, 51]. Due to the different training design, it may be possible that the inflammatory reactions start at different times.

Like shown in Fig. 5, IL-6 and IL-10 serum concentrations were increased by the 6 days repeated training load protocol. A short-term chronic treatment with ALA could counteracted the increase (Fig. 5). These effects are in agreement to findings in patients with metabolic diseases [39–41, 43]. In such patient’s treatment with ALA also reduces the serum concentrations of inflammation markers such as IL-6, or TNF-α. In Fig. 5 it is shown that also in healthy young athletes, the training induced increase of IL-6 is inhibited by short-term chronic ALA supplementation whereas in the control group a significant increase of IL-6 could be observed after 7 days (*p* = .005*). Even if no group difference could be detected, a small to medium effect between the two groups could be determined at T2 (IL-6: d = .587, IL-10: d = .428) and are confirm previous studies. However, the inhibition of IL-6 should be interpreted in relation to the context. A reduction of inflammatory processes is a major goal in treatment strategies for various diseases in order to improve the quality of life and reduce long-term side effects [56–61]. However, with respect to physical activity the induction of muscle damage and inflammation is an important stimulus to activate molecular mechanisms responsible for an adaptation of the physiological processes, and at the end for the training effect [2, 54, 62–64]. It is an important challenge to find the right balance between training intensity and the resulting skeletal muscle damage and inflammation. A complete suppression of inflammatory processes in response to physical activity, by anti-inflammatory drugs like ibuprofen or diclofenac has been demonstrated to counteract training adaptations [65, 66]. On the other hand, a too strong stimulation of the inflammatory processes by training in association with muscle damage, can even lead to rhabdomyolysis [67–70]. In the process of inflammation mediated training adaptations, macrophage formation plays a decisive role and can be divided into two phenotypes [71, 72]. Inflammation-promoting M1 macrophages (eg. IL-6) migrate first into the tissue and contribute decisively to the degradation of necrotic tissue. At the same time, they stimulate the proliferation of myoblasts. This is followed by the immigration of anti-inflammatory M2 macrophages (eg. IL-10) [63], which balance the previously necessary inflammatory reactions and thus promote the recovery of muscle tissue [13–15]. However, when M1 macrophages are suppressed directly, M2 macrophages are not expressed either. This effect could also be observed in T2 (+ 7 d). However, in the ALA-group not only the training induced increase of IL-6, also the increase of IL-10 serum concentration was inhibited by ALA (IL-6 pre: 5.1 pg/ml (SD 4.7), post: 7.8 pg/ml (SD 10.4); IL-10 pre: 8.5 pg/ml (SD 9.3), post: 11.0 pg/ml (SD 15.5). A typical anti-inflammatory activity of a substance normally results in a suppression of pro-inflammatory cytokines like IL-6 but an increase of the serum concentrations of anti-inflammatory cytokines like IL-10 [63]. This profile could be observed recently in previous studies using protein/carbohydrate supplementation as a pro-regenerative intervention intensive endurance training [45]. However, in this study a chronic six-day training protocol was used to simulate a training camp. The main goal of a training camp is not to improve performance in the shortest possible time, but to increase the load capacity and competition hardness [70]. The suppression of IL-6 and IL-10 could also be reproduced in this study design by short-term chronic ALA supplementation. If all effects on muscle damage, inflammation and performance are considered separately, the individual effects are rather small to moderate. However, the individual parameters in sport are very closely related, so that they cannot be considered individually, but only in combination in the overall context. Even if no clear group difference could be determined, a trend towards the effect of ALA in sport can be identified on the basis of the same courses of the independent parameters. Under this aspect it is remarkable that the suppression of training-induced IL-6 and IL-10 by ALA is very effective and this is reproducible in the reduced loss of performance. In competitive sports these small differences can be very decisive for victory and defeat.

One of the major pharmacologic activities of ALA discussed, are a high anti-oxidative capacity [27, 39, 73]. Therefore, in this study effects of ALA on markers for oxidative stress were analysed. As shown in Fig. 6, the biomarker for oxidative stress, oxLDL, was not increased by the chronic training protocol indicating that this training protocol may not be suitable to induce chronic oxidative stress and/or that our participants are too well trained and compensate this stress. Therefore, it was also not possible in this experimental design to investigate potential anti-oxidative effects of ALA. Our training protocol includes resistance, but also endurance training. With the focus on anti-oxidative capacity of ALA in training, it would be more suitable to use a high intensity endurance training protocol for 6 days. However, the focus of this study was on skeletal muscle recovery and maintenance of performance. Based on the observations, it cannot be excluded that ALA also has positive effects in sports through antioxidant activities, but this aspect seems to be less relevant in this study design. Nevertheless, follow up studies should focus also on the effects of ALA treatment in the context of physical activity, especially in long-term treatment over several weeks.

In summary, the data of our study indicate that ALA may support skeletal muscle recovery in scenarios involving chronic training and treatment and prevent the training related loss in performance. In contrast, we did not find any significant effects of ALA in acute training and treatment scenarios. These results suggest also that ALA treatment needs to be chronically to result in training-supporting effects. This is also indicated by our biomarkers for skeletal muscle damage and inflammation.

Regarding possible beneficial effects of ALA in training adaptation it is important to figure out that a district-level of skeletal muscle damage is required to activate molecular mechanisms that lead to training adjustments [74]. Chronic application of ALA may negatively influence this adaptation if the training goal is to improve performance. However, long-term training planning and periodization is based not only on performance improvement, but also on maintaining and improving the hardness of competition. Especially sports, which have competitions over several days, must maintain their performance as good as possible. In this context, hard training weeks or training camps are often held to simulate the competition phases. In this context, chronic ALA supplementation may be useful to maintain performance. Our study design could show first indications and trends that ALA can have a positive effect on maintaining performance and reducing muscle damage and inflammation.. In which sports or scenarios ALA can be usefully applied must be determined in further studies. We conclude that short-term chronic ALA supplementation can support muscle recovery effects and prevents the training induced loss of performance in a specific training or competition phase, in very well-trained athletes. However, for general recommendations, further investigations in different training scenarios and in athletes with different performance, are necessary, also to identify underlying molecular mechanisms. The application time of ALA may also need to be extended in order to achieve more distinct courses and results.

### Limitations/outlook

Due to the limitations in the study protocols, it is not yet possible to make final statements regarding the training supporting effects of a chronic supplementation with ALA in athletes and healthy, very well-trained individuals. There is a clear need for further investigations. In such investigations, ALA supplementation should be done over longer periods of time to find out, how ALA affects training outcome. Furthermore, no clear statements can currently be made whether there are different effects of ALA in strength or endurance training. Based on the available data from clinical trials in chronic diseases [24–26, 28–30], it could be assumed that ALA could possibly achieve a better effect in endurance than in resistance training. Possible gender differences in the effect of ALA should also be taken into account in future studies to further specify the application profile.

Due to the anti-oxidative function of ALA as a radical scavenger that this could be a further advantage in long-term endurance training scenario or in competition situations known to induce high levels of anti-oxidative stress like marathon running.

## Supplementary Information

**Additional file 1 **

## Data Availability

The raw data can be viewed upon request.

## Abbreviations

1RM BS: 1 repetition maximum back squat
ALA: Alpha lipoic acid
CK: Creatine kinase
HDL: High density lipoprotein
IkB: Inhibitor kappa B
IL-6: Interleukin 6
IL-10: Interleukin 10
LDL: Low density lipoprotein
MAPK: Mitogen-activated protein kinase
MYO: Myoglobin
NFkB: Nuclear factor kappa B
oxLDL: Oxidative low density lipoprotein
SD: Standard deviations
TNF-α: Tumor necrosis factor alpha
