# Supplementary material for: The effects of alpha lipoic acid on muscle strength recovery after a single and a short-term chronic supplementation - a study in healthy well-trained individuals after intensive resistance and endurance training
Source: J Int Soc Sports Nutr. 2020 Dec 1;17:61. doi: 10.1186/s12970-020-00389-y (PMC7708149; doi:10.1186/s12970-020-00389-y)
Supplement: Supplementary file 1 — Additional file 1 [file 12970_2020_389_MOESM1_ESM.pdf]

Table 3A: ALA-group individual change of 1RM back squat, absolute and relative

Subjects	1RM back squat ALA T0	1RM back squat ALA T2(+7d)	Baseline T0	relative change T2(+7d)
P1	110	115	1,00	1,045
P2	145	140	1,00	0,966
P3	105	105	1,00	1,000
P4	135	125	1,00	0,926
P5	155	150	1,00	0,968
P6	200	200	1,00	1,000
P7	170	175	1,00	1,029
P8	110	115	1,00	1,045
P9	130	130	1,00	1,000
P10	140	135	1,00	0,964
P11	95	95	1,00	1,000
P12	110	110	1,00	1,000
P13	105	110	1,00	1,048
P14	105	110	1,00	1,048
P15	105	110	1,00	1,048
P16	130	135	1,00	1,038
P17	140	150	1,00	1,071
Mean	128,82	130,00	1,00	1,012
SD	27,09	26,35	0,00	0,038

Table 3B: PL-group individual change of 1RM BS, absolute and relative

Subjects	1RM back squat PL T0	1RM back squat PL T2(+7d)	Baseline T0	relative change T2(+7d)
P1	115	115	1,00	1,000
P2	135	130	1,00	0,963
P3	100	100	1,00	1,000
P4	130	130	1,00	1,000
P5	150	150	1,00	1,000
P6	210	195	1,00	0,929
P7	185	175	1,00	0,946
P8	100	105	1,00	1,050
P9	130	130	1,00	1,000
P10	140	140	1,00	1,000
P11	90	90	1,00	1,000
P12	105	105	1,00	1,000
P13	115	105	1,00	0,913
P14	105	105	1,00	1,000
P15	110	100	1,00	0,909
P16	120	125	1,00	1,042
P17	140	135	1,00	0,964
Mean	128,24	125,59	1,00	0,983
SD	30,29	27,22	0,00	0,039

Table 4. All results.

	Group	T0	T1	T2	Time effect (T0/T1)	Time effect (T0/T2)	Time*group effect (T0/T1)	Time*group effect(T0/T2)	effect size T7d
**1RM BS**	ALA	128.8 (SD 27.9)	125.9 (SD 29.3)	130.0 (SD 27.2)	.053	.320	.992	.028#	.164
**(in kg) n=17**	PL	128.2 (SD 30.3)	125.3 (SD 28.0)	125.6 (SD 27.2)	.051	.032*	.992	.028#	.164
**Myoglobin**	ALA	35.4 (SD 9.1)	51.0 (SD 32.9)	50.5 (SD 30.3)	.045*	.059	.581	.130	.467
**(in ng/ml) n=16**	PL	33.8 (SD 9.4)	53.2 (SD 32.9)	71.1 (SD 52.4)	.002*	.008*	.581	.130	.467
**Creatinkinase**	ALA	290.6 (SD 232.1)	519.0 (SD 436.5)	464.1 (SD 250.3)	.014*	.426	.119	.136	.212
**(in U/L) n=14**	PL	217.1 (SD 146.1)	597.0 (SD 422.5)	534.6 (SD 392.6)	.000*	.005*	.119	.136	.212
**IL6 (in pg/ml)**	ALA	5.1 (SD 4.7)	2.7 (SD 0.6)	7.8 (SD 10.4)	.092	.249	.795	.107	.587
**n=16**	PL	6.0 (SD 6.1)	2.8 (SD 1.0)	16.8 (SD 18.1)	.050	.005*	.795	.107	.587
**IL10 (in pg/ml)**	ALA	8.5 (SD 9.3)	3.5 (SD 2.2)	10.2 (SD 14.5)	.136	.554	.479	.959	.428
**n=17**	PL	7.2 (SD 8.5)	3.4 (SD 1.3)	18.6 (SD 23.0)	.289	.035*	.479	.959	.428
**HDL (in mg/dl)**	ALA	50.0 (SD 7.6)	---	47.2 (SD 7.1)	---	.077	---	.270	.041
**n=16**	PL	49.4 (SD7.3)	---	50.3 (SD 7.9)	---	.326	---	.270	.041
**LDL (mg/dl)**	ALA	104.9 (SD 26.0)	---	100.9 (SD 26.4)	---	.246	---	.730	.063
**n=17**	PL	107.2 (SD 20.3)	---	102.4 (SD 20.9)	---	.266	---	.730	.063
**oxLDL (in U/L)**	ALA	65.9 (SD 21.3)	---	54.0 (SD36.5)	---	.339	---	.270	.083
**n=16**	PL	69.3 (SD 20.2)	---	49.8 (SD 61.4)	---	.074	---	.270	.083

Time effects were marked with *; Time x group effects were marked with #.

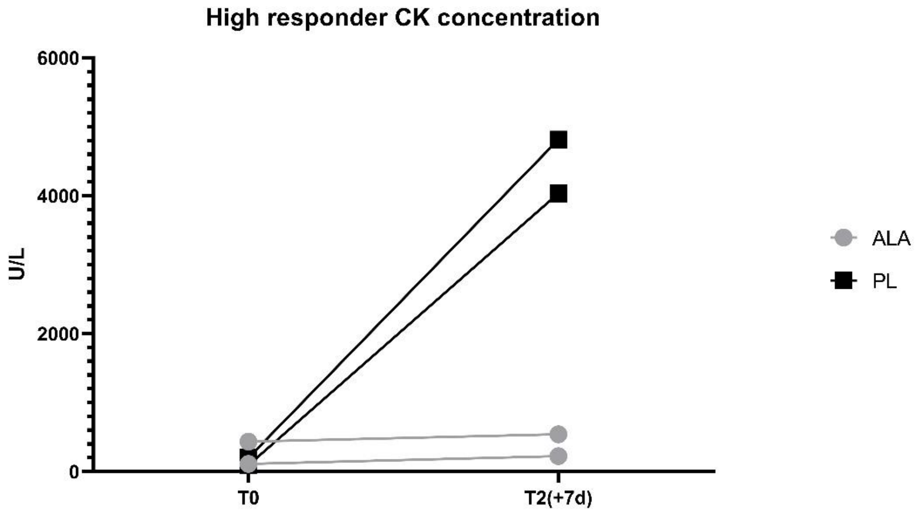

Figure 4 A2 two individual high responder CK concentration
